# Perceived Caregiver Strain, 3- and 18-Month Poststroke, in a Cohort of Caregivers from the Life after Stroke Trial (LAST)

**DOI:** 10.1155/2022/2619893

**Published:** 2022-03-31

**Authors:** Birgitta Langhammer, Hege Ihle Hansen, Bent Indredavik, Torunn Askim

**Affiliations:** ^1^Department of Health, Oslo Metropolitan University, Sunnaas Rehabilitation Hospital, Oslo, Norway; ^2^Department of Medicine, Vestre Viken Hospital Trust, Bærum Hospital, Oslo, Norway; ^3^Department of Medicine, Oslo University Hospital, Ullevål, Oslo, Norway; ^4^Department of Neuroscience, Faculty of Medicine, NTNU Norwegian University of Science and Technology, Trondheim, Norway

## Abstract

**Aim:**

To gain more knowledge of caregiver strain in the Life After Stroke Trial (LAST) population.

**Methods:**

This is a substudy of the LAST study, including all caregivers' reports of perceived Caregiver Strain Index (CSI) at inclusion and 18-month follow-up irrespective of group allocation. The checklist “STROBE Statement—Checklist of items that should be included in reports of cohort studies” was used. Caregivers to adults (age ≥18 years), here defined as the person living with, a patient with a first-ever or recurrent stroke, community dwelling, with modified Rankin Scale (mRS) <5 and no serious comorbidities, was invited to fill out the Caregiver Strain Index at three months (10–16 weeks) poststroke. Domains indicating differences of change in perceived strain in the total sample were analyzed in a linear regression analysis.

**Results:**

Caregiver strain (*n* = 147) varying from 5% to 27% was reported by the caregivers at baseline and between 2% to18% at 18-month follow-up. The items indicating the highest level of strain at baseline and 18 months were as follows: “Care giving is confining,” “There have been changes in personal plans,” “There have been emotional adjustments,” and “I feel completely overwhelmed.” The samples were divided into age groups 0–79 years and 80–100 years, indicating a higher strain on the caregiver for persons 80–100 years at 18 months.

**Conclusion:**

Caregiver strain was relatively low both at baseline and at 18-month follow-up. Main caregiver strains were reported in terms of a sense of confinement, a tendency of emotional strain, and the altering of plans at both time points. Depression was one of the main explanatory factors for the perceived caregiver strain. The perception of caregiver strain was higher in age groups 80–100 years than age groups 0–79 years.

## 1. Introduction

Most of the long-term care for chronically disabled elders is provided by informal caregivers. Caregiving has been recognized as an activity that both confers perceived benefits and produces caregiver strains. The caregiver strain is a complex construct, usually defined by its impact and consequences for the caregiver [1]. It can be an important way of coping with stress, contributing to “meaning-based coping” by the caregiver [2]. But informal caregivers, family members, friends, or neighbors, are unpaid individuals who may provide as much as 90% of the in-home long-term care without compensation [3]. For many caregivers, caregiving is a job that is stress filled, overwhelming, and isolating. Emotional, psychological, physical, and economic impacts of caregiving have been used to define and assess caregiver strain [1]. Caregivers may be prone to depression, grief, fatigue, and changes in social relationships and/or, experience physical health problems of their own [4].

Caregiver strain has been broadly classified into two categories: objective and subjective caregiver strains [5].

Objective caregiver strain refers to its physical effect on the other household members for day-to-day tasks they undertake for the “patient,” such as the time invested by the caregiver on helping, supervising, and feeding him or her [6]. It also includes their experiences of family disturbance and relationship problems, and loss of employment and/or leisure because of the caregiving tasks [7].

The perceived subjective caregiver strain relates to the psychological, social, and emotional impacts that caregivers may experience [8]. Experiences of changes of roles, feelings of guilt, shame, embarrassment, and self-blame in families of people with chronic illness have been revealed in several studies [9–11].

The perceived caregiver strain is universal, independent of the type of chronic disease. However, stroke caregivers have to cope with variable levels of cognitive deficits and/or physical disability that imply considerable objective and subjective caregiver strain [12]. In addition, stroke caregivers are possibly older persons and may be experiencing challenges in their own health [13], which cause levels of anxiety and depression above normal [14].

Despite this knowledge, little attention is paid to caregivers of persons with stroke [15] and the phenomenon of being an informal caregiver to a home-dwelling family member suffering from stroke is little explored.

The aim of this substudy was to gain more knowledge of caregiver strain including the caregiver's perception of domains of strain poststroke.

## 2. Materials and Methods

This is a substudy of the Life After Stroke Trial (LAST) study of caregiver strain [16]. The LAST study was a multicentre, pragmatic, single-blinded, randomized controlled trial performed at two centres in Norway (Trondheim University Hospital and Bærum Hospital), in close collaboration with the primary healthcare service in the municipalities of Trondheim, Asker, and Bærum to evaluate a longitudinal follow-up of coaching and physical activity, and details of this randomized controlled trial are provided in reference [16].

The results of this trial were neutral, so this substudy entails all caregivers irrespective of group allocation. The material was analyzed as one sample of caregivers. Caregiver strain was evaluated with a questionnaire screening at two time points at 3- and 18-month poststroke in the total cohort of caregivers. The STROBE Statement—Checklist was used (“STROBE statement Strengthening the reporting of observational studies in epidemiology”).

Ethical approval for the trial was granted by the Central Regional Committee for Medical and Health Ethics (REC no. 2011/1428).

### 2.1. Subjects

Caregivers to adults (age ≥18 years), here defined as the person living with, a patient with a first-ever or recurrent stroke, community dwelling, with modified Rankin Scale (mRS) <5 and no serious comorbidities, was invited to fill out the Caregiver Strain Index at three months (10–16 weeks) poststroke. Data were collected indirectly, and questionnaires have been delivered to caregivers, husbands, or wives, if they met with their partner at test, both at inclusion and at 18 months follow-up. Alternatively, it was sent with the participant for delivery at home with cohabitant caregivers. Answers were sent back in prepaid envelopes. Sex and marital status but no other descriptive data of the caregivers were recorded. Only data from respondents and the person with stroke are included in the analyses.

### 2.2. Baseline Characteristics

Baseline variables of the patient population were age, sex, and marital status. In addition, Barthel Index (BI), Modified Rankin Scale (mRS), Gait speed, six-minute walk test (6MWT), Hospital Anxiety and Depression Scale (HADS), and Mini-Mental State Examination (MMSE) were used to describe their functional, physical, cognitive, and emotional levels at baseline [16]. In addition, the caregivers were invited to fill out Caregiver Strain Index (CSI) at baseline and at 18-month follow-up, which is the outcome reported in this substudy.

### 2.3. Caregiver Strain Index

The Caregiver Strain Index (CSI) was developed in 1983 [17] and is an easy-to-use tool that can quickly screen for caregiver strain in long-term caregivers. The tool has 10 questions that measure different domains of strain related to care provision [17]. The replies are categorized as yes/1 and no/0 answers, for example: “I am going to read a list of things that other people have found to be difficult. Would you tell me whether any of these apply to you? Sleep is disturbed (e.g., because … is in and out of bed or wanders around at night”) [18]. It may be used to assess individuals of any age who have assumed the caregiving role of an older adult. The internal reliability coefficient is reported for the CSI in 1983 (0.86), as well as a cutoff score of 7 or more positive items, indicating higher levels of stress underpinning the need for further assessment [17]. Minimal detectable change is reported as ±2.8 [19]. The Caregiver Strain Index has been reported to be reproducible, but only moderately responsive, when measuring caregiver strain perceived by caregivers of stroke patients [17, 19].

### 2.4. Analysis

Descriptive data of the persons with stroke whose caregivers answered the Caregiver Strain Index are presented as mean (SD), frequencies, and percentages. Caregivers' perceived caregiver strain is presented in frequencies and percentages.

Domains indicating differences of change in perceived strain in the total sample were further analyzed in a linear regression analysis. The items were entered as dependent factors, with age, sex, BI, 6MWT, HADS-D, and mRS as independent factors. The independent variables were tested individually for their associations and included in the multiple linear regression analysis if they reached the predefined statistical levels of *p* < 0.2. Residuals of the regression models were tested for normality using the Shapiro–Wilks test. The alpha level was set to *p* < 0.05.

## 3. Results

Table 1 lists the characteristics of the patient population divided into groups of perceived strain at both 3 and 18 months, as well as dropouts. Total CSI forms received at baseline were 269 and 174 at 18-month follow-up, with a total of 147 completing CSI at both baseline and 18 months. The majority answering the questionnaire were caregivers of male stroke participants at baseline (*n* = 161, 60%) and 18 months (*n* = 106, 61%). The respondents were cohabiting/married to the person with stroke and of approximately the same age (Table 1).

Caregiver strain (*n* = 147) was reported as low as 5% (answering “yes, financial strain”) and up to 29% (“I feel completely overwhelmed”) by the caregivers at baseline (Table 2) [17]. The items indicating the highest level of strain at baseline were as follows: “Caregiving is confining” (24%), “There have been changes in personal plans” (27%), and “There have been emotional adjustments” (26%) and “I feel completely overwhelmed” (29%). At 18-month follow-up, the same tendency remained but now varying with a slightly lower perception of caregiver strain with 2% “yes” answers (“Caregiving is a physical strain”) to 18% (“I feel completely overwhelmed”) (Table 2). Items indicating most strain at this time point were as follows: “There have been changes in personal plans” (15%), “There have been emotional adjustments” (17%), and “I feel completely overwhelmed” (18%). In general, CSI scores indicated no change/less perceived strain from baseline to 18 months (Table 3). Twelve percent of the participants had a minimal detectable change ±2.8 of total score, of which 6% was a change for the better (19).

In this study, including 269 caregivers of persons with stroke, 40% were females (*n* = 108) and 60% males (*n* = 161) at baseline. One hundred and ninety-seven persons with stroke (*n* = 197) were 0–79 years of age, 38% females (*n* = 74) and 62% males (*n* = 123) and (*n* = 72) were ≥80 years of age, 34 women (47%), and 38 men (53%).

The sample divided into age 0–79 years and ≥80 years indicated a higher strain on the caregiver for persons ≥80 years, especially at 18 months (Table 4). The domains “Caregiving is inconvenient” both at baseline and 18 months, “Caregiving is confining,” and “There have been emotional adjustments” at 18 months indicated a perceived higher strain in the caregiving (for a person ≥80 years of age). In addition, financial strain in the same group showed a tendency to increase, at the 18-month follow-up. When divided into gender groups, female caregivers for persons with stroke ≥80 years reported worse sleep at baseline and 18 months. Male caregivers reported higher emotional strain in persons with stroke ≥80 years at baseline and 18 months.

In terms of change between 3 and 18 months, two domains were reported significantly changed (Table 2) and were further analyzed in a regression analysis. In the domain, “there have been other demands on my time,” the main explanatory independent factor was depression (HADS-D, *β* = 0.212, *p*=0.01) with *R*^2^ = 0.08 at 18 months. The second domain, “I feel completely overwhelmed,” the explanatory independent factors at baseline, 3 months, were age (*β* = −0.235, *p*=0.001), BI (*β* = −0.153, *p*=0.0025), 6 MWT (*β* = −0.166, *p*=0.003) and HADS-D (*β* = 0.168, *p*=0.01) with an *R*^2^ = 0.137. At 18 months, HADS-D was the main explanatory independent factor (*β* = 0.196, *p*=0.019) with *R*^2^ = 0.074 for the same domain.

## 4. Discussion

In this sample of relatively highly independent persons with stroke, included 3-month poststroke, the caregiver strain, defined as >7 positive answers on total CSI, indicates a slight decrease in perceived caregiver strain over time in the total sample. The main domains of caregiver strain were an inconvenience, sense of confinement, tendency to need emotional adjustments, and altering of plans at both time points (Table 2). There was, however, a perception of higher strain in age groups ≥80 years of age, which is in line with earlier studies on the subject [13, 14].

The responses “Caregiving is confining” and “There have been emotional adjustments” both indicated higher strain in the assumed older caregivers than with the “younger” 0–79 years of age (Table 4). This may be related to the caregiver's health, which may suffer due to not being able to (confining) maintain physical and psychological social interactions with “others” (participation) because of duties within the home and in relation to the person with stroke [20, 21]. It may also be an interaction with the persons with stroke's worsening health/disability, since physical functioning in general deteriorated between baseline and 18-month follow-up [16, 22]. The tendency of emotional strain has been reported in caregivers of persons with stroke in terms of depression but also in terms of perceived quality of life [20]. The emotional strain in the caregivers has also been related to the level of disability of the person with stroke [20, 22].

In the acute and postacute stages, the tendency of total life crisis, both for the person with stroke and also for their next of kin, has been well described [11]. The last item “I feel completely overwhelmed” was reported relatively high at baseline and slightly less at 18 months, in line with those reports [12]. In addition, there was no difference between 0–79 years or ≥80 years of age, indicating that the global perception of a severe life crisis was present in the subacute phase but subdued over time (Table 2). The reason for this may be twofold: it may reflect that the persons with stroke were relatively high functioning and had no cognitive deficits (Table 1). Another reason may be that the municipal services are well developed in Norway, relieving financial and physical strains. This relatively low perception of caregiver strain may be a reflection of the fact that services for formal care are satisfactory for this group of relatively high functioning persons with stroke and their caregivers [23].

However, there were tendencies for an increased caregiver strain with higher age in the 18-month follow-up, indicating a change for the worse, which may be related to both the person with stroke and their caregiver's health combined with general ageing (Table 4) [24].

The item “financial strain” was reported by 5% in the age group ≥80 years and 9% in the group 0–79 years at baseline, again perhaps mirroring a well-functioning welfare state with a functioning social security system for the majority [20]. On the other hand, at 18 months, the financial strain in the group ≥80 was reported to have increased (Table 4). This may indicate that those caregivers/persons with stroke encounter increased financial problems in a longitudinal perspective perhaps related to health issues and disability, which may lead to the reduced working capacity for both the person with stroke and their caregiver [20, 24].

Interestingly, the explanatory factor for the domains changing significantly for the worse in terms of perceived strain was depression in the person with stroke, in line with other studies [15]. Depression has also been related to the reported feelings of being overwhelmed and suffering emotional strain [12].

### 4.1. Limitations

There are several limitations to this substudy. First, the main study was a randomized controlled study but not a prospective cohort including all persons with stroke and their next of kin. The results can only be transferable to persons with stroke with the inclusion criteria of the trial and their married or cohabiting partners. The recruitment of caregivers was in most cases indirect via the person with stroke and the sample may not be representative for a general population of caregivers to persons with stroke. In addition, a relatively high drop-out rate of informants of caregiver strain was registered at 18 months, which makes it challenging to compare the findings from baseline and 18-month follow-up. Furthermore, any in-depth description of caregivers is lacking. However, the sample was large compared to other studies on the subject. The informants were recruited from urban areas, not representing the rural and more sparsely populated areas, which limits the generalizability of the results. The results may be indicative for groups with minor to moderately disabled persons with stroke and their partners and may serve as indicators for the needs in this group of caregivers.

## 5. Conclusion

In this sample of relatively independent persons with stroke, including 3 months poststroke, caregiver strain was relatively low both at baseline and at 18-month follow-up. Main caregiver strains were reported in terms of a sense of confinement, a tendency for emotional strain, and the alteration of plans at both time points. Depression was one of the main explanatory factors for the perceived caregiver strain. The perception of caregiver strain was higher in age groups ≥80 years than age groups 0–79 years.

### 5.1. Relevance to Clinical Practice

The items indicating the highest level of strain at baseline and 18 months were related to psychological adjustments (“Caregiving is confining” (24% vs. 13%), “There have been emotional adjustments” (26% vs. 17%), “I feel completely overwhelmed” (29% vs. 18%)), and practical issues (“There have been changes in personal plans” (27% vs. 15%)). This indicates a need for supportive psychological and practical services for caregivers during and after rehabilitation, which nurses have an opportunity to initiate to alleviate caregiver strain.

## Figures and Tables

**Table 1:** Baseline demographic and clinical characteristics in mean (m) and standard deviation (SD). In persons with stroke divided into groups of reported change in Caregiver Strain Index (CSI), in frequencies (*n*) and percentages (%), between 3 and 18 months.

	Improved (*n* = 7)	Minor/no difference baseline/18 mo (*n* = 130)	Deteriorated (*n* = 10)	No data CSI (*n* = 233)
Age (m; SD)	67.3 (9.6)	70.9 (10.5)	73.2 (10.1)	72.7 (11.7)
Gender females/males (*n*, %)	2 (29%)/5 (71%)	51 (39%)/79 (61%)	5 (50%)/5 (50%)	91 (39%)/142 (61%)
Civil status single/cohabitant (*n*, %)	3 (43%)/4 (57%)	36 (28%)/94 (72%)	2 (20%)/8 (80%)	65 (28%/168 (72%)
Barthel Index (m; SD)	94.29 (6.1)	96.87 (7.64)	95.5 (6.4)	95.2 (8.9)
Barthel Index 18 mo (m; SD)	96.43 (4.8)	96.56 (9.86)	87.48 (19.0)	84.66 (30.9)
6MWT^1^ baseline (m; SD)	457.5 (55.1)	445.93 (147.2)	352.5 (178.4)	389.23 (153.4)
6MWT 18 mo (m; SD)	453. 20 (115.2)	455.16 (124.8)	379.0 (187.8)	361.29 (198.8)
Gait speed baseline m/s (m; SD)	1.22 (0.6)	1.48 (0.6)	1.46 (0.6)	1.25 (0.5)
Gait speed 18 mo·m/s (m; SD)	1.57 (0.7)	1.48 (0.6)	1.23 (0.6)	0.81 (0.7)
MMSE^2^ baseline (m; SD)	26.86 (2.9)	28.12 (2.3)	28.1 (2.1)	27.9 (2.3)
MMSE 18 mo (m; SD)	28.7 (1.9)	27.97 (2.5)	26.6 (5.5)	27.4 (3.6)
HADS^3^ baseline (m; SD)	3.0 (2.3)	2.55 (2.6)	4.22 (5.3)	3.69 (3.1)
HADS 18 mo (m; SD)	2.86 (3.6)	3.0 (2.82)	5.8 (5.9)	3.81 (3.0)
TMT-A^4^ baseline s (m; SD)	61.5 (33.5)	57.58 (28.6)	92.5 (60.0)	64.53 (36.9)
TMT-B^5^ baseline s (m; SD)	2.86 (3.6)	143.92 (70.4)	196.83 (95.8)	138.51 (77.4)
TMT-A 18 mo s (m; SD)	51.29 (23.1)	63.31 (42.4)	72.56 (54.5)	62.2 (36.8)
TMT-B 18 mo s (m; SD)	154.57 (92.3)	140.06 (76.9)	122.80 (54.1)	125.09 (68.5)
mRS baseline (m; SD)	1.3 (1.3)	1.34 (1.1)	1.57 (1.4)	1.51 (1.1)
NIHSS^6^ total baseline	1.2 (1.6)	1.5 (2.5)	1.5 (1.6)	1.5 (2.2)

^1^6MWT = 6 minute walk test, ^2^MMSE = minimal mental status evaluation, ^3^HADS = hospital anxiety and depression scale, ^4^TMT-A = trail making test A, ^5^TMT-B = trail making test B, ^6^NIHSS = the national institutes of health stroke scale. mo, months.

**Table 2:** All reported answers of Caregiver Strain Index (CSI) items at baseline 3 and 18-month follow-up. The yes/no answers are reported in frequencies (*n*) and percentages (%). In addition, a total score of more than 7 positive answers is reported reflecting a high burden of caregiver strain.

CSI items	Baseline 3 months	18 months
My sleep is disturbed	(*n* = 269)	(*n* = 175)
Yes	42 (16%)	20 (11%)
No	227 (84%)	155 (89%)
Caregiving is inconvenient	(*n* = 266)	(*n* = 170)
Yes	32 (12%)	14 (8%)
No	234 (88%)	156 (82%)
Caregiving is a physical strain	(*n* = 264)	(*n* = 174)
Yes	12 (5%)	4 (2%)
No	252 (95%)	170 (98%)
Caregiving is confining	(*n* = 266)	(*n* = 174)
Yes	63 (24%)	23 (13%)
No	203 (76%)	151 (87%)
There have been family adjustments	(*n* = 266)	(*n* = 172)
Yes	41 (15%)	17 (10%)
No	225 (85%)	155 (90%)
There have been changes in personal plans	(*n* = 265)	(173)
Yes	72 (27%)	26 (15%)
No	193 (73%)	147 (85%)
There have been other demands on my time	(*n* = 267)	(*n* = 173)
Yes	46 (17%)	12 (7%)
No	221 (83%)	161 (93%)
There have been emotional adjustments	(*n* = 264)	(*n* = 174)
Yes	69 (26%)	29 (17%)
No	195 (74%)	145 (83%)
Caregiving is a financial strain	(*n* = 267)	(*n* = 174)
Yes	15 (6%)	7 (4%)
No	252 (94%)	167 (96%)
I feel completely overwhelmed	(*n* = 266)	(*n* = 172)
Yes	78 (29%)	31 (18%)
No	188 (71%) (*n* = 242)	141 (82%) (*n* = 122)
(i) Total score of 7 positive answers	*n* = 10 (4%)	*n* = 15 (12%)

**Table 3:** Caregivers divided into groups, based on level of perceived strain, and comparisons of change in Caregiver Strain Index domains between 3 and 18 months, in frequencies (*n*) and percentages (%).

	Improved	No/minor change	Deteriorated
My sleep is disturbed *n* = 147	7 (4.8%)	130 (88.4%)	10 (6.8%)
Caregiving is inconvenient *n* = 140	12 (8.6%)	121 (86%)	7 (5%)
Caregiving is a physical strain *n* = 143	1 (0.7%)	140 (97.9%)	2 (1.4%)
Caregiving is confining *n* = 142	18 (12.7%)	115 (81%)	9 (6.3%)
There have been family adjustments *n* = 140	9 (6.4%)	122 (87%)	9 (6.3%)
There have been changes in personal plans *n* = 139	17 (12.2%)	111 (79.9%)	11 (7.9%)
There have been other demands on my time *n* = 143	14 (9.8%)	127 (88.8%)	2 (1.4%)
There have been emotional adjustments *n* = 142	12 (8.5%)	123 (86.6%)	7 (4.9%)
Caregiving is a financial strain *n* = 143	4 (2.8%)	134 (93.7%)	5 (3.5%)
I feel completely overwhelmed *n* = 142	27 (19%)	103 (72.5%)	12 (8.5%)
Total score *n* = 142	36 (25%)	88 (62%)	18 (13%)

**Table 4:** All answers of the Caregiver strain index (CSI) at baseline and 18-month (mo) follow-up in groups divided in age groups 0–79 years and >80 years, with yes/no answers in answer frequencies (*n*) and percentages (%).

	Baseline	18 mo	Baseline	18 mo
Items (*n*; %)	Age 0–79 *n* = 197	Age 0–79 *n* = 139	Age ≥ 80 *n* = 72	Age ≥ 80 *n* = 36

*My sleep is disturbed*				
Yes	29 (15%)	13 (9%)	13 (18%)	7 (19%)
No	168 (85%)	126 (91%)	59 (82%)	29 (81%)

*Caregiving is inconvenient*				
Yes	18 (9%)	5 (4%)	14 (19%)	9 (26%)
No	176 (91%)	130 (96%)	58 (81%)	26 (74%)

*Caregiving is a physical strain*				
Yes	9 (5%)	3 (2%)	3 (4%)	1 (3%)
No	186 (95%)	135 (98%)	66 (96%)	35 (97%)

*Caregiving is confining*				
Yes	46 (24%)	12 (9%)	17 (24%)	11 (31%)
No	149 (76%)	126 (91%)	54 (76%)	25 (69%)

*There have been family adjustments*				
Yes	26 (13%)	10 (7%)	15 (21%)	7 (21%)
No	168 (87%)	128 (93%)	57 (79%)	27 (79%)

*There have been changes in personal plans*				
Yes	50 (26%)	16 (12%)	22 (31%)	10 (28%)
No	145 (74%)	121 (88%)	48 (69%)	26 (72%)

*There have been other demands on my time*				
Yes	34 (17%)	9 (7%)	12 (17%)	3 (8%)
No	162 (83%)	128 (93%)	59 (83%)	33 (92%)

*There have been emotional adjustments*				
Yes	53 (27%)	18 (13%)	16 (24%)	11 (69%)
No	143 (73%)	120 (87%)	52 (76%)	25 (31%)

*Caregiving is a financial strain*				
Yes	9 (5%)	7 (5%)	6 (9%)	35 (100%)
No	188 (95%)	132 (95%)	64 (91%)	—

*I feel completely overwhelmed*				
Yes	59 (30%)	24 (18%)	19 (27%)	7 (20%)
No	137 (70%)	113 (82%)	51 (73%)	28 (80%)

## Data Availability

Data are available on request to the authors.
